# The self-efficacy in patient-centeredness questionnaire – a new measure of medical student and physician confidence in exhibiting patient-centered behaviors

**DOI:** 10.1186/s12909-015-0427-x

**Published:** 2015-09-15

**Authors:** Robert Zachariae, Maja O’Connor, Berit Lassesen, Martin Olesen, Louise Binow Kjær, Marianne Thygesen, Anne Mette Mørcke

**Affiliations:** 1 Unit for Psychooncology and Health Psychology, Department of Oncology, Aarhus University Hospital and Department of Psychology, Aarhus University, Bartholins Allé 9, DK8000 Aarhus, Denmark; 2 Center for Teaching and Learning, School of Business and Social Science, Aarhus University, Fuglsangs Alle 4, 8210 Aarhus, Denmark; 3 Centre for Health Sciences Education, Aarhus University, INCUBA, Palle Juul-Jensens Boulevard 82, bld. B, 8200 Aarhus, Denmark; 4 Faculty of Health, University of Southern Denmark, J.B. Winsløws Vej 19, 3, 5000 Odense, Denmark

## Abstract

**Background:**

Patient-centered communication is a core competency in modern health care and associated with higher levels of patient satisfaction, improved patient health outcomes, and lower levels of burnout among physicians. The objective of the present study was to develop a questionnaire assessing medical student and physician self-efficacy in patient-centeredness (SEPCQ) and explore its psychometric properties.

**Methods:**

A preliminary 88-item questionnaire (SEPCQ-88) was developed based on a review of the literature and medical student portfolios and completed by 448 medical students from Aarhus University. Exploratory Principal Component analysis resulted in a 27-item version (SEPCQ-27) with three underlying self-efficacy factors: 1) Exploring the patient perspective, 2) Sharing information and power, and 3) Dealing with communicative challenges. The SEPCQ-27 was completed by an independent sample of 291 medical students from 2 medical schools and 101 hospital physicians.

**Results:**

Internal consistencies of total and subscales were acceptable for both students and physicians (Cronbach’s alpha (range): 0.74–0.95). There were no overall indications of gender-related differential item function (DIF), and a Confirmatory Factor Analysis (CFA) indicated good fit (CFI = 0.98; NNFI = 0.98; RMSEA = 0.05; SRMR = 0.07). Responsiveness was indicated by increases in SEPCQ scores after a course in communication and peer-supervision (Cohen’s *d* (range): 0.21 to 0.73; *p*: 0.053 to 0.001). Furthermore, positive associations were found between increases in SEPCQ-scores and course-related motivation to learn (medical students) and between SEPCQ scores and years of clinical experience (physicians).

**Conclusions:**

The final SEPCQ-27 showed satisfactory psychometric properties, and preliminary support was found for its construct validity, indicating that the SEPCQ-27 may be a valuable measure in future patient centered communication training and research.

## Background

It is generally agreed that the quality of the patient-clinician relationship is important for providing and receiving excellent care, for the healing process, and for health-related outcomes [1–3], and that patient-centeredness is a central aspect of high quality health care [4]. Several terms, including “patient-centered communication”, “patient-centered care”, and “patient-centered medicine” are often used interchangeably, and it has been proposed that the term “patient-centeredness” should be reserved to describe a general moral philosophy of health care with three core attributes: a) consideration of patients’ needs, perspectives, and individual experiences, b) provision of opportunities to patients to participate in their care, and c) enhancement of the patient-clinician relationship [4, 5]. Patient-centered communication can be defined as the processes and communicative behaviors exhibited by clinicians that promote these core values. One operational definition includes: a) Eliciting and understanding the patient’s perspective – concerns, ideas, expectations, needs, feelings, and functioning, b) understanding the patient within his or her unique psychosocial context, c) reaching a shared understanding of the problem and its treatment with the patient that is concordant with his or her values, and d) helping patients to share power and responsibility by involving them in choices to the degree that they wish [5]. Other similar definitions are available [6, 7].

Over the years, a growing body of research has explored the possible influence of patient-centered communication on various patient outcomes. Several systematic reviews, both narrative [8–12] and quantitative, using either simple vote counting [13] or meta-analytic approaches [14–17] focusing on various types communicative behaviors, different types of clinicians, and a variety of patient populations have been published. The most frequently studied patient outcomes are patient satisfaction and adherence to treatment, with results of meta-analyses generally showing medium effect sizes. Associations with patient health outcomes, on the other hand, are less frequently studied. The available studies also provide support for the effectiveness of communication skills training in the form of improved communicative behaviors of clinicians [18] and higher patient satisfaction [19], whereas the effects in terms of patient outcomes [20–22] are mixed. While most of the studies have focused on physicians, there is also some research on the promotion of communicative skills among medical students, and the results of a meta-analysis [23] suggests that certain teaching methods, e.g. small group teaching, yield relative large effects. As the foundation of patient-centered approaches among future physician, including the attitude towards the physician-patient relationship and basic communicative skills, is likely to be laid in medical school, more research in this important areas is needed.

Patient-centered behaviors have also been found associated with physician well-being. It has, for example, been found that physicians with high levels of stress and burnout engage less in developing the relationship with the patient [24, 25] and that training physicians the skills of mindful practice reduce their level of burnout, improve their job satisfaction, and their levels of empathy; factors that are associated with the ability to engage with patients in a patient-centered manner [26]. These findings suggest that the mental health of physicians’ is associated with their communicative skills and their ability to focus effectively and empathically in their interactions with patients [27]. Taken together, the relatively sparse literature suggests a complex bi-directional relationship between physician burnout, communication skills, and patient satisfaction.

Concurrent with the growing evidence, the development and maintenance of skills in patient-centered communication has become an important element in medical education, making it increasingly relevant to assess different aspects of patient-centeredness, both as outcomes of communication training and as predictors of patient and physician outcomes. Although communication-related competencies are often assessed with interaction analysis systems evaluating observed verbal and nonverbal communicative behaviors [28], such assessments are complex and time consuming. There is therefore a need for practical and valid questionnaire-based methods to assess competencies in patient-centeredness.

With respect to the attainment of knowledge, attitudes, and skills, there is increasing attention towards the role that the person’s beliefs play in this process. Self-efficacy, a key term in social cognitive theory, has been defined as confidence or “*beliefs in one’s capabilities to organize and execute the courses of action required to produce given attainments*” [29, 30], and a growing body of research has confirmed that self-efficacy is likely to affect the individual’s behavior in such key aspects as the tasks and approaches they choose, their exertion, perseverance, and performances. In research of physician communication skills and communications skills training, outcomes have often been assessed as levels and changes in the physician’s confidence in various communication skills, e.g. general interviewing skills [31, 32], communicating bad news to oncology patients [33], behavioral counseling skills for multiple risk factor modification [34], and in dealing with mental health problems presented in general practice [35]. The outcomes, however, have usually been assessed with various ad-hoc-developed measures, including a frequently used 9-item measure [32], and - to the best of our knowledge - a validated and psychometrically sound instrument to measure self-efficacy in exhibiting all core aspects of patient centered behaviors and attitudes for use in medical education and physician-patient communication research has so far not been available. Our aim was therefore to develop a Self-Efficacy in Patient Centeredness Questionnaire and to provide preliminary evidence for its reliability and validity in medical students and physicians.

## Methods

### Procedure and participants

**Fig. 1:**
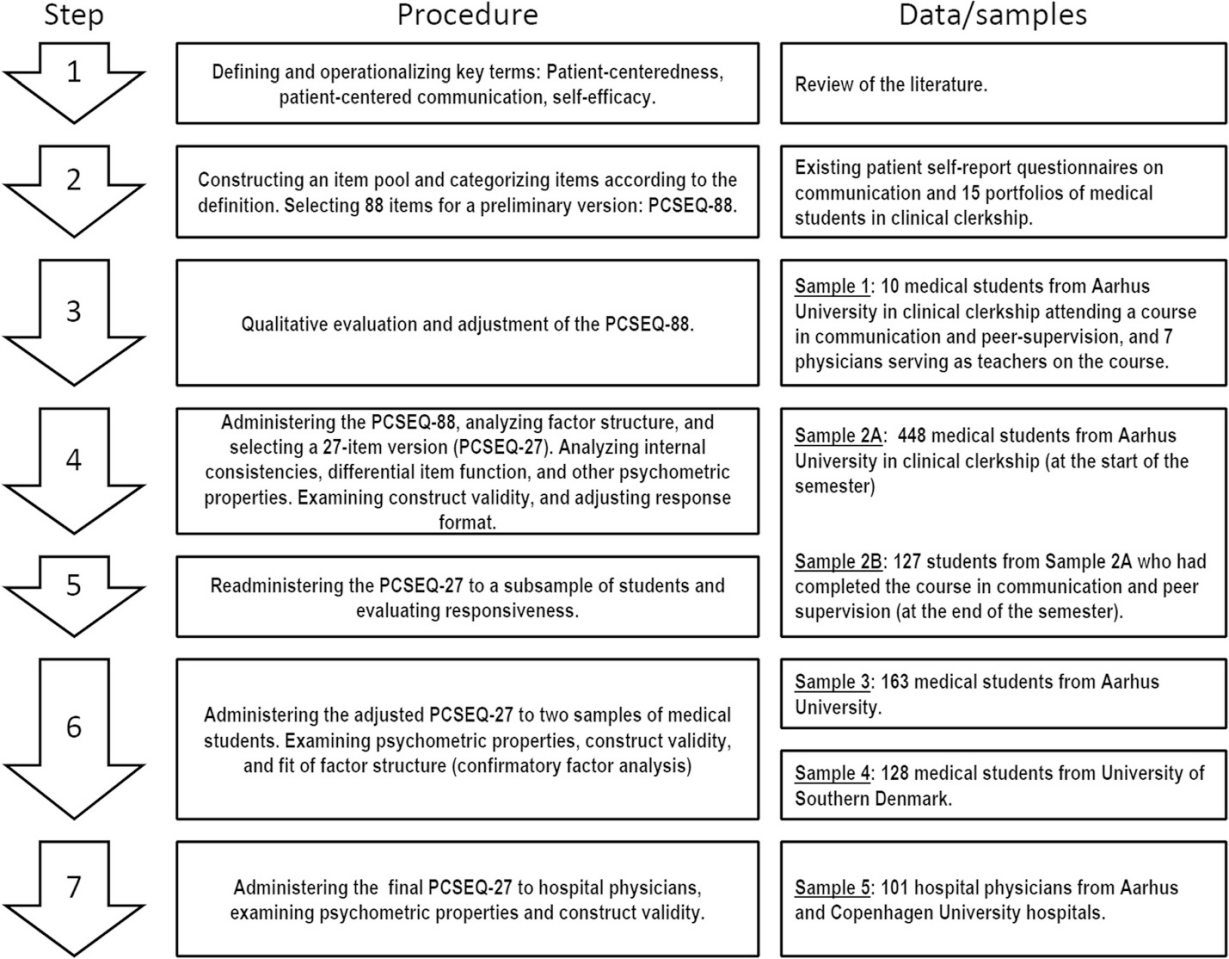
Overview of the development and validation procedure for the Patient-Centeredness Self-Efficacy Questionnaire (SEPCQ)

The development and preliminary validation of the Self-Efficacy in Patient Centeredness Questionnaire (SEPCQ) was guided by the quality criteria developed by the Scientific Advisory Committee of the Medical Outcomes Trust (MOT) [36] and conducted in seven steps involving five samples totaling 749 medical students and 108 physicians (Fig. 1).

### Step 1: defining patient-centeredness self-efficacy

Based on a review of the literature, we chose a general definition of patient-centeredness as exhibiting three core attributes: 1) “*Considering the patients’ needs, wants, perspectives, and individual experiences*”, 2) “*offering patients opportunities to provide input into and participate in their care*”, and 3) “*enhancing the partnership and understanding in the patient-physician relationship*” [5]. This definition was supplemented with definitions presented by Stewart and colleagues [6], which resulted in an operational definition of patient-centered communication as being characterized by the following five physician behaviors: 1) *Eliciting, understanding, and validating the patient’s perspective* (e.g. concerns, feelings, expectations, values). 2) *Viewing the patient’s health status, disease, and symptoms from a bio-psycho-social perspective* (e.g. life history, developmental issues, employment, community), 3) *Reaching a shared understanding of the patient’s problem and its treatment*, taking into consideration the patient’s priorities, the aims of treatment, and the roles of patient and physician, 4) *Helping the patient share power* by offering meaningful involvement in choices relating to his or her health. 5) *Enhancing the patient-doctor relationship* by focusing on compassion, healing, sharing power, quality-of-life, self-awareness, and transference.

Following Bandura’s definition [30], medical student or physician patient-centeredness *self-efficacy* was defined as his or her confidence in his or her ability to exert each particular behavior in a manner so that the patient would perceive it according to its underlying intention. Items would thus be worded as: “*I am confident that I am able to make the patient experience me as* (specific behavior covered by the item)”.

### Step 2: constructing an item pool

A large initial item pool was then constructed based on: a) items from five available published patient questionnaires [37–41] and b) communication-related issues and behaviors described in 15 portfolios produced by medical students in clinical clerkship at university hospital clinics who attended a course on communication and peer-supervision. Four of the authors independently selected relevant behaviors and issues and categorized them according to the five behavioral characteristics listed above or – if needed – into additional categories. A preliminary pool of 88 items was then negotiated with items grouped into 6 initial domains: 1) Exploring the patient perspective (21 items), 2) Viewing the patient’s problem from a bio-psycho-social perspective (e.g. background, life situation) (9 items), 3) Establishing a shared understanding of the disease, examinations, and treatment (9 items), 4) Helping the patient to influence his/her care (15 items), 5) Enhancing the physician/medical student-patient relationship (20 items), and 6) Self-reflection and dealing with communicative challenges (14 items).

### Step 3: SEPCQ construction and preliminary evaluation

The first 88-item version of the SEPCQ (SEPCQ-88) was then constructed. A 7-point Likert scale was used with “0” (to a very low degree) and “6” (To a very high degree) as endpoints. The middle score “3” was anchored with “Neither/nor”. A general instruction was developed emphasizing that the questions covered neither the actual behavior nor the desirability of the behavior but rather the respondent’s *confidence* in exhibiting each particular behavior so that patients will experience the described behavior as intended. (See instructions in Table 1). Two authors then evaluated the instructions, response categories, and wordings of items using the QAS-99 Question Appraisal System [42] leading to some minor adjustments. The SEPCQ-88 was then administered to 10 students and 7 physicians (Sample 1), who were interviewed about the relevance, acceptability, and understandability of the instructions and items, leading to some final adjustments.

**Table 1:** The Self-Efficacy in Patient Centeredness Questionnaire (SEPCQ-27): Instructions, item wordings, factor loadings, and differential item function (DIF)

Items: I am confident that I am able to …	Factor 1: Exploring the patient perspective	Factor 2: Sharing information and power	Factor 3: Dealing with communicative challenges	DIF-MH	DIF-IRT
				ETC.-category ^c^	K-index ^d^
1. Make the patient feel that I am genuinely interested in knowing what he/she thinks about his/her situation	0.69	0.18	0.21	A	0.15
2. Make the patient feel that I have time to listen	0.66	0.28	0.21	A	0.13
3. Recognize the patient’s thoughts and feelings	0.69	0.18	0.30	A	0.06
4. Be attentive and responsive	0.74	0.25	0.15	A	0.13
5. Be aware of when the patient is scared or concerned	0.62	0.26	0.32	A	0.14
6. Treat the patient in a caring manner	0.77	0.26	0.08	A	0.04
7. Make the patient experience me as empathetic	0.81	0.19	0.13	A	0.08
8. Make the patient feel that he/she can talk with me about confidential, personal issues	0.64	0.28	0.27	A	0.04
9. Show a genuine interest in the patient and his/her situation	0.80	0.23	0.18	A	0.10
10. Focus on compassion, care and symptomatic treatment, when there is no curative treatment	0.71	0.25	0.18	A	0.06
11. Record a complete medical history	0.19	0.56	0.15	A	0.13
12. Reach agreement with the patient about the treatment plan to be implemented	0.27	0.60	0.24	A	0.14
13. Advise and support the patient in making decisions about his/her treatment ^b^	0.34	0.60	0.27	A	0.10
14. Ensure that the patient makes his/her decisions on an informed basis	0.25	0.71	0.27	A	0.18
15. Explain the diagnosis and treatment plan to the patient so that he/she understands	0.17	0.73	0.10	A	0.12
16. Explain things so that the patient feels well-informed	0.35	0.76	0.15	A	0.07
17. Inform the patient about the expected side effects, so the patient understands them	0.30	0.65	0.22	A	0.13
18. Explain how the treatment works or is expected to work	0.12	0.82	0.13	A	0.10
19. Explain how the treatment is likely to affect the patient's condition, so that the patient understands	0.25	0.79	0.16	A	0.06
20. Explain the treatment procedures, so that the patient understands them	0.18	0.83	0.13	A	0.07
21. Accept when there is no longer curative treatment for the patient	0.10	0.23	0.63	A	0.20
22. Be aware of when my own feelings affect my communication with the patient	0.22	0.14	0.70	A	0.11
23. Deal with my own emotional reactions when the situation is difficult for me	0.16	0.11	0.68	A	0.28
24. To maintain the relationship with the patient when he/she is angry	0.22	0.14	0.65	A	0.11
25. To stay focused on what is best for the patient if there is a professional disagreement about the diagnosis and treatment ^b, c, d^	0.10	0.34	0.62	B	0.34
26. Avoid letting myself be influenced by preconceptions about the patient ^d^	0.25	0.10	0.68	A	0.32
27. Separate my personal views from my approach in the professional situation	0.21	0.11	0.69	A	0.03

Instructions: In the following, a number of statements describing different aspects of how physicians and medical students can relate to and communicate with patients are presented. Please read each statement carefully and judge how *confident* you are in your ability to relate to and communicate with patients in the manner described in the statement. Please answer all questions and provide your best assessment of how confident you are that you will be able to behave in the way described in the statement. Please answer as honestly and sincerely as possible. Remember that each question must be answered based on how confident you are that you will be able to *make the patient experience* the particular behavior - not the extent to which you would like to be able to engage in the behavior^a^

^a)^English translation-backtranslation: The original Danish item wordings were translated independently into English by two English-speaking persons, and following a backtranslation, a final English version was negotiated

^b)^Two items (13 and 25) did not meet the primary criterion (difference between the highest and second highest loading ≥ 0.30), but were retained in the model on the basis of content analysis. The remaining 25 items met both criteria: Loading ≥ 0.50; difference between highest and second highest loading ≥ 0.30

^c)^Mantel-Haenszel Chi-Square Differential item function (DIF) analysis: Negligible gender-related DIF (A) for all items except item 25 (moderate DIF = B) in favor of men (Zieky, 1993)

^d)^IRT DIF-analysis: K-index (Kumagai, 2012) for item 25 and 26 exceeded the criterion of 0.30 with possible gender-bias in favor of men (item 25) and women (item 26)

### Step 4–7: psychometric evaluation procedure

The SEPCQ-88 was first completed by Sample 2A (see Fig. 1 for brief description of samples) and completed again a second time by Sample 2B. The psychometric properties of the full SEPCQ-88 were evaluated, leading to a revised 27-item version (SEPCQ-27) with changes made to the scoring format (from a 7-point to a 5-point Likert scale) (see Results for further details). The SEPCQ-27 was then administered to two new student samples (Sample 3 and 4) and a sample of hospital physicians (Sample 5) and subjected to further evaluation.

#### Additional measures

All students were asked to report age, gender, and current semester. Physicians were asked to report age, gender, department, current position, year of graduation from medical school, and whether they had attended the standard patient-communication training course offered by the Danish Medical Association (DMA).

All students completed the Medical Student Well-Being Index (MSWBI) [43], a 7-item measure of medical student distress.

Students from Sample 2A who were scheduled to attend the course in communication and peer-supervision also completed a Course-Related Motivation to Learn questionnaire (CRMTL) (10 items) and a Course-related Self-Efficacy questionnaire (CRSE) (5 items), both adapted from the Motivated Strategies for Learning Questionnaire (MSLQ) [44]. The CRMTL assessed the students’ motivation to take the course. Sample item: “*In a course like this, I prefer exercises that really challenge me, so that I can learn new things*”, and the CRSE measured expectancy regarding how well he/she would do on the course. Sample item: “*I expect to do exceptionally well on this course*”. Internal consistencies (Cronbach’s Alpha) in the present sample were 0.86 (CRMTL) and 0.84 (CRSE).

Sample 3 and 4 also completed a 12-item version of the Marlowe Crowne Social Desirability Scale (MCSDS) [45] as a measure of possible response bias, i.e., the likelihood of reporting behaviors and responses that are culturally sanctioned but relatively unlikely. The MCSD-12 has previously displayed moderate, but acceptable, internal consistencies (KR-20: 0.69-0.71) in Danish university students (*N* = 457) (unpublished data) and showed satisfactory internal consistency in the present sample (KR-20: 0.73).

## Analytical strategy

### Identifying the factor structure

The possible underlying factor structure was explored as recommended [46] with a series of Principal Components Analyses with Varimax rotation and Eigenvalues >1 as criterion. Factor loadings were required to be >0.50 and the difference between the highest and second-highest factor loading at least 0.30. Items with differences in factor loadings between 0.20 and 0.29 were reviewed as possible items. A subsequent series of Principal Components analyses restricted to extract 7, 6, 5, 4, and 3 factors (fixed factor analyses) were then conducted. The factors were identified using the following criteria: a) Consistency in factor loadings across the six analyses and b) the item content should consistently reflect the same underlying factor. A final Principal Component Analysis with the selected items was then conducted to verify the model.

### Reliability

Score distributions, ranges, means, and standard deviations of each item, the sum scores of the three derived subscales, and the total score were reviewed. Internal consistencies (Cronbach’s alpha) were calculated together with the baseline to14-16 weeks test-retest correlation for students who had completed the SEPCQ both before and after a course in communication and peer-supervision (Sample 2B).

### Differential Item Function (DIF)

If DIF is present for an item, this could indicate measurement bias, i.e. that individuals from different groups with the same underlying ability (latent trait) differ in their likelihood to respond in a certain way to an item [47]. DIF analysis was used to evaluate possible gender-related measurement bias of the 27 selected items using both the Mantel-Haenszel (MH) Chi-Square [48] and an Item-Response-Theory (IRT)-based approach [49, 50]. The MH approach was based on the following criteria: 1) The MH Chi-Square (MH-CHI), 2) the MH Common Log-Odds Ratio (MH-LOR), 3) The standard error of MH-LOR (LOR SE), 4) the Standardized MH Log-Odds Ratio (LOR Z), and 5) the Breslow-Day Chi-Square (BD) (critical values: 3.84; *p* < 0.05). The Combined Decision Rule (CDR) suggests acceptable results if none of the above parameters reach statistical significance. The ETS Categorization Scheme represents consensus about DIF size (A = negligible, B = moderate, C = large) [51]. The IRT-based approach was based on the K-index for polytomous data [52]. If K > ((N categories–1) × 0.1), a large DIF is assumed. The lowest response categories (0–3) had many instances of “0” frequency, and as IRT does not allow “0” frequency, the scores were recoded: 0-3 = 1, 4 = 2, 5 = 3, and 6 = 4. The K-index cutoff was therefore ((4–1) × 0.1) = 0.3. Items with a K-index > 0.3 were assumed to have DIF.

### Confirmatory factor analysis (CFA)

The fit of the three-factor SEPCQ-27 version was evaluated with CFA of the data collected from the independent Sample 4 from a different medical school. Five commonly used goodness-of-fit indices were used to evaluate the model with Robust Maximum Likelihood estimation: The Satorra-Bentler Scaled Chi-Square (S-B-Chi-Square), the Comparative Fit Index (CFI), the Non-Normed Fit Index (NNFI), the Root Mean Square Error of Approximation (RMSEA), and the Standardized Root Mean Square Residual (SRMR) [53, 54]. The model was interpreted as providing a reasonable good fit given a statistically significant S-B chi-square, CFI and NNFI > 0.95, RMSEA < 0.06, and SRMR < 0.08.

Construct validity was explored by examining associations with background variables (age, gender, experience) and scores on the CRMTL, CRSE, and MSWBI. SEPCQ scores were expected to be positively correlated with: a) Older age and female gender (students) and b) older age, more experience, and previous communication training (physicians). We also expected c) that higher levels of distress (MSWBI) would be associated with lower SEPCQ-scores, and d) that physicians would present higher scores than students. Testing responsiveness, we expected e) that completing the course in communication and peer-supervision would be associated with increased post-course SEPCQ scores. We also expected f) that higher pre-course CRMTL and CRSE scores would be independently associated with greater increases in SEPCQ scores.

### Ethics

All data were collected anonymously. In Denmark, studies involving questionnaire-based data only are not subjective to ethical approval by the Regional Ethics Committees.

## Statistical software

The majority of the analyses were conducted with IBM SPSS Statistics version 21. The confirmatory Factor analysis (CFA) was conducted with LISREL, version 8.80. Mantel-Haenszel Chi-Square DIF analyses were conducted with DIFAS version 4.0. [48], and IRT-based DIF analyses with EasyDIF version 0.1.6 [52].

## Results

### Step 4 and 5

A total of 520 out of approximately 660 7^th^ to 10^th^ semester students (Sample 2A) were present at the lectures where the SEPCQ-88 was handed out. A total of 448 (86.2 %) questionnaires with complete or almost complete data were returned. Number of missing items ranged from 2.2 % to 2.8 %.

#### Factor structure

The first Principal Components analysis converged after 17 iterations with 14 factors explaining 64.2 % of the variation. A Scree plot indicated a flattening of the slope with more than 3 factors, and the subsequent (fixed factor) Principal Component Analyses (data not shown) resulted in a preliminary 27-item 3-factor model, which was then subjected to a final Principal Components analysis. The model converged after 5 iterations explaining 58.9 % of the variation. A content analysis suggested the following three underlying dimensions of Self-Efficacy in Patient Centeredness: Factor 1: Exploring the patient perspective (10 items from the initial domains 1 and 5), Factor 2: Sharing information and power (10 items from domains 3 and 4), and Factor 3: Dealing with communicative challenges (7 items from domain 6). Only one item from the initial domain 2 was included (in Factor 2). Mean factor loadings for Factor 1–3 were 0.71, 0.71, and 0.66 respectively, and the average differences between the highest and second highest loadings were 0.46, 0.46, and 0.44. The final 3-factor model is shown in Table 1. Sample characteristics are shown in Table 2.

**Table 2:** The Self-Efficacy in Patient Centeredness Questionnaire. First 88-item version (SEPCQ-88) and final 27-item (SEPCQ-27): Sample characteristics and descriptive data

Total N: 857	Score range	Sample 1	Sample 2A	Sample 2B	Sample 3	Sample 4	Sample 5
Participants		Medical students + physicians	Medical students				Physicians
N	-	10 + 7	448	(127)	163	128	101
Questionnaires:	-	SEPCQ-88	SEPCQ-88, MSWBI, CRMTL, CRSE	SEPCQ-88^a^	SEPCQ-27, MCSD-12, CRMTL, CRSE	SEPCQ-27, MCSD-12	SEPCQ-27
Semester	-	8	Mixed	8	8	Mixed	NA
Men, N (%)	-	5 (29.4 %)	90 (29.7 %)	38 (30.0 %)	52 (31.9 %)	31 (24.2 %)	32 (31.7 %)
Age, Mean (SD)	-	-	24.8 (2.1)	24.6 (1.8)	24.7 (1.6)	25.6 (3.9)	41.7 (10.4)
Years of experience, Mean (SD)	-	-	-	-	-	-	13.9 (10.8)
SEPCQ-Factor 1, Mean (SD)	0-40	-	27.8 (7.4)	29.0 (6.3)	18.6 (4.2)	21.3 (5.4)	32.4 (4.5)
SEPCQ-Factor 2, Mean (SD)	0-40	-	23.9 (7.9)	26.8 (6.5)	15.2 (5.1)	15.6 (6.3)	31.9 (4.9)
SEPCQ-Factor 3, Mean (SD)	0-28	-	12.6 (6.0)	14.3 (5.4)	7.0 (4.0)	8.3 (4.0)	19.7 (3.7)
SEPCQ-27 Total, Mean (SD)	0-108	-	64.2 (18.0)	70.1 (15.0)	40.9 (10.6)	45.1 (13.5)	83.9 (11.4)
MCSD-12, Mean (SD)	0-12	-	-	-	1.5 (0.1)	2.2 (0.2)	-
CRMTL, Mean (SD)	10-70	-	49.2 (9.6)	-	47.6 (10.4)	-	-
CRSE, Mean (SD)	5-35	-	22.9 (5.1)	-	22.0 (4.9)	-	-
MSWBI, Mean (SD)	0-7	-	1.6 (2.1)	-	1.3 (1.6)	-	-

SEPCQ- Factor 1 (Exploring the patient perspective); SEPCQ- Factor 2 (Sharing information and power); SEPCQ- Factor 3 (Dealing with communicative challenges); MCSD-12 (Marlowe-Crowne Social Desirability Scale, 12-item Short Version); CRMTL (Course-Related Motivation to Learn); CRSE (Course-Related Self-Efficacy); MSWBI (Medical Student Well-Being Index)

^a)^SEPCQ data recoded from the original 7-point Likert scale format (0–6) into a 5-point Likert scale format (0–4) to allow for comparison of SEPCQ-88 and SEPCQ-27 data

#### Psychometric properties of SEPCQ-27

The score distributions of each item, each factor, and the total scale were inspected. Item response frequencies for the three lowest scoring categories (0–2) were low with average cumulative item response frequencies (scoring category 0 + 1 + 2) of 2.9 %, 5.7 %, and 17.6 % for Factor 1, 2, and 3 respectively. Furthermore, when inspecting the score distributions of the factors and total scales, Factor 1 and 2 appeared relatively skewed in the positive direction.

Internal consistencies (Cronbach’s Alpha) ranged between 0.83 and 0.94 (see Table 3), correlations between subscales ranged between 0.50 and 0.60, and subscale-to-total scale correlations ranged between 0.78 – 0.86 (see Table 4). The 14–16 week retest correlations for Factor 1, 2, 3, and total SEPCQ-27 were 0.62, 0.47, 0.69, and 0.87 respectively (*p* < 0.01) (Sample 2B).

**Table 3:** The Self-Efficacy in Patient Centeredness Questionnaire (SEPCQ-27). Internal consistencies (Cronbach’s Alpha)

		Medical students		Physicians
	Items	Sample 2A	Sample 3 + 4	Sample 5
N		448	291	101
SEPCQ- Factor 1	10	0.93	0.85	0.90
SEPCQ- Factor 2	10	0.92	0.90	0.93
SEPCQ- Factor 3	7	0.83	0.74	0.84
SEPCQ-27 Total	27	0.94	0.92	0.95

SEPCQ- Factor 1 (Exploring the patient perspective); Factor 2 (Sharing information and power); Factor 3 (Dealing with communicative challenges)

**Table 4:** The Self-Efficacy in Patient Centeredness Questionnaire (SEPCQ-27). Factor inter-correlations and correlations with age, course-related motivation to learn (CRMTL), course-related self-efficacy (CRSE), social desirability (MCSD-12), and age and years of experience (physicians)

	SEPCQ - factor 1			SEPCQ - factor 2			SEPCQ - factor 3			SEPCQ-27 total		
	Medical students		Phys.	Medical students		Phys.	Medical students		Phys.	Medical students		Phys.
Sample	2A^a^	3 + 4	5	2A^a^	3 + 4	5	2A^a^	3 + 4	5	2A^a^	3 + 4	5
SEPCQ-Factor 2	0.60**	0.59**	0.65**	-	-	-	-	-	-	-	-	-
SEPCQ- Factor 3	0.54**	0.55**	0.52**	0.50**	0.58**	0.74**	-	-	-	-	-	-
SEPCQ-27 Total	0.86**	0.85**	0.84**	0.86**	0.88**	0.92**	0.78**	0.80**	0.84**	-	-	-
CRMTL^b^	0.53**	-	-	0.21	-	-	0.40**	-	-	0.49**	-	-
CRSE^b^	0.55**	-	-	0.34**	-	-	0.46**	-	-	0.58**	-	-
MSWBI	−0.14**	-	-	−0.18**	-	-	−0.25**	-	-	−0.22**	-	-
MCSD-12	-	0.27** -	-	-	0.06	-	-	0.20** -	-	-	0.20** -	-
Age^c^	0.07	0.15*	0.14	0.08	0.17**	0.36**	0.16**	0.21**	0.49**	0.12*	0.18**	0.36**
Experience (yrs)	-	-	0.20*	-	-	0.40**	-	-	0.50**	-	-	0.36**
Age (yrs) (Adjusted for experience)^d^	-	-	−0.09	-	-	−0.01	-	-	0.09	-	-	−0.01
Experience (yrs) (Age adjusted)^d^	-	-	0.17	-	-	0.19	-	-	0.16	-	-	0.41**

SEPCQ-Factor 1: Exploring the patient perspective; SEPCQ-Factor 2: Sharing information and power; SEPCQ-Factor 3: Dealing with communicative challenges; MCSD-12: Marlowe-Crowne Social Desirability Scale, 12-item short version; CRMTL: Course-Related Motivation to Learn; CRSE: Course-Related Self-Efficacy; MSWBI: Medical Student Well-Being Index

^a)^Correlations for Sample 2A (*N* = 463) calculated with recoded (0–4) response categories to increase comparability with Sample 3 + 4 (*N* = 291)

^b)^CRMTL, CRSE: Results only for 8^th^ semester students prior to taking the course in communication and peer-supervision

^c)^Wider age distribution in Sample 3 + 4 than 2A

^d)^Partial correlations

*)*p* < 0.05; ** *p* < 0.01

DIF analysis was conducted with women (*N* = 358) as reference group and men (*N* = 90) as focal group using the MH Chi-Square approach. Based on the Combined Decision Rule (CDR) and the ETS Categorization Scheme [51], almost all items had negligible gender-related DIF (category A). The exception was item 25 (“*I am confident that I am able to stay focused on what is best for the patient if there is a professional disagreement about the diagnosis and treatment*”) which showed moderate item bias (B) in favor in men, i.e. the item was “easier” for men than women. The IRT-based DIF analysis generally confirmed the results of the MH Chi-Square approach. No K-index values for any items in Factor 1 and 2 exceeded the criterion of 0.3. Two items in Factor 3: item 25 (see above) and item 26 (“*I am confident that I am able to avoid letting myself be influenced by preconceptions about the patien*t”) showed indications of DIF with values slightly exceeding 0.30 (K = 0.34 and 0.32). The Item Characteristic Curves of men and women indicated that item 25 showed a tendency towards favoring men, while item 26 appeared to favor women. The average K-index values for factors 1, 2, and 3 were 0.09, 0.11, and 0.20 respectively. The results of the DIF analyses are shown in Table 1.

#### Construct validity

One-way ANOVA’s showed that 10^th^ semester students (Sample 2A), who had not had any communication training, had lower scores than the remaining students. A total of 41 students (10.0 %) with non-Danish ethnicity exhibited lower scores than Ethnic Danish students (statistically non-significant). T-tests for independent samples indicated that men (*N* = 90) had slightly higher scores than women (*p* < 0.05) for Factor 2, Factor 3, and the Total scale, but not Factor 1 (mean differences: 2.4 to 4.6) (data not shown).

As seen in Table 4, small, but positive, correlations were found between age and SEPCQ scores (r = 0.07 – 0.16), with correlations with Factor 3 and SEPCQ total scores reaching statistical significance (*p* < 0.05). Students with higher levels of psychological distress (MSWBI) tended to have lower SEPCQ scores. In students, who were scheduled to take the course in communication and peer-supervision, both CRMTL and CRSE were positively associated with higher scores on the SEPCQ at the beginning of the semester. SEPCQ-scores increased for students who also completed the SEPCQ after completing the course, with changes corresponding to small-to-medium effect sizes (Cohen’s *d*) [55] of 0.21, 0.38, 0.45, and 0.73 for Factor 1, 2, 3, and Total scores respectively. Changes reached statistical significance (*p* < 0.001) for Factor 2, 3, and Total scores but not Factor 1 (*p* = 0.053).

Multiple linear regression analyses with post-course SEPCQ scores as dependent variables and CRMTL or CRSE as predictors, while adjusting for pre-course SEPCQ-scores, showed that pre-course CRMTL was a statistical significant predictor of post-course SEPCQ-scores for Factor 1 (Beta:0.21; *p* = 0.02); Factor 2 (Beta:0.21; *p* = 0.02), and SEPCQ Total scores (Beta:0.25; *p* = 0.005). A near-significant trend was found for Factor 3 (Beta:0.16; *p* = 0.052). None of the results for CRSE reached statistical significance (Beta: 0.07–0.18; *p*:0.06–0.40).

#### Adjustments

Although the Principal Component and DIF analyses indicated that item 25 might be problematic, the item was retained as the values (factor loading difference, the ETS, and K-index) only slightly exceeded the criteria, and the content appeared to reflect an important issue related to patient-communication-related challenges. The major concern was that for several items, primarily in factor 1 and 2, only very few responders chose the lowest scoring categories (0–2). The low item difficulties were hypothesized to be due to a) the high number of response categories and b) the mid-point anchor of 3 = “neither/nor”, which risked being interpreted as “indifference”. The response format was therefore changed to a 5-point Likert scale (0–4) with no mid-point anchor and endpoints anchored as “To a very low degree” and “To a very high degree”.

### Step 6

The results obtained for the revised SEPCQ-27 when administered to Sample 3 and 4 are shown in Table 2. Both subscale and total scores now appeared normally distributed with similar means and medians (Means: 19.7, 15.4, 7.5, 42.7; Medians: 19, 16, 7, 43) and low skewness (−0.09, −0.12, 0.51, 0.12) and kurtosis (−0.16, 0.016, 0.41, 0.34) for the combined Sample 3 and 4. Internal consistencies, between-subscale correlations, and correlations between subscales and SEPCQ-27 total scores were generally similar to those found for Sample 2A (Tables 3 and 4). Small, but statistically significant, positive correlations were found with age. In Sample 3 and 4, women scored slightly higher than men on Factor 1 (mean difference = 2.0; *p* < 0.02) and SEPCQ-27 Total (3.0; *p* < 0.05). No gender differences were found for Factor 2 and 3. When exploring the possibility of response bias with the MCSD-12, small, but significant, positive correlations with social desirability were found for Factor 1, 3, and total SEPCQ scores (Table 4).

#### Confirmatory Factor Analysis (CFA)

When conducting a CFA with the SEPCQ-27 data from the independent sample of SDU students, the results for the chosen fit indices were: S-B Chi Square (441.2; *p* < 0.001); CFI = 0.98 (criterion > 0.95); NNFI = 0.98 (criterion > 0.95); RMSEA = 0.05 (90 % CI: 0.04 – 0.06) (criterion: < 0.06), and SRMR = 0.07 (criterion: 0.08). As a significant S-B Chi Square was expected, and the remaining 4 indices indicated good fit [53], no attempts were made to explore the fit of alternative models.

### Step 7

A convenience sample of 101 physicians (response rate: 44 %) (Sample 5) also completed the SEPCQ-27. Gender, age, years of experience, and mean SEPCQ-27 scores are shown in Table 2. Internal consistencies were acceptable (0.84 – 0.95) (Table 3) and correlations between subscales and between subscales and SEPCQ-27 total scores were generally similar to those found for medical students (Table 4). Scores on Factor 2, 3, and total SEPCQ-27 - but not Factor 1 - showed statistical significant correlations with both age and years of experience. Years of experience correlated with age (r = 0.90), but when adjusting for years of experience, the correlations between SEPCQ scores and age (−0.09 to 0.09) no longer reached statistical significance. In contrast, when adjusting for age, all correlations with years of experience remained positive and reached statistical significance for SEPCQ-27 total scores (Table 4). Physicians exhibited statistically significantly higher scores than medical students on all SEPCQ scores (*p* < 0.001) (data not shown), and physicians who had participated in the DMA communication course had statistically significant higher scores on Factor 1, 2, and SEPCQ total (Cohen’s *d*: 0.55, 0.45, 0.48; *p*: 0.007, 0.03, 0.02), but not Factor 3 (*d* = 0.19; *p* = 0.30). No gender differences were found (data not shown).

## Discussion

After having defined the concepts and possible categories of patient-centeredness self-efficacy, having developed an initial item pool, and evaluated items and instructions on the basis of established quality criteria, the first 88-item and the revised 27-item version of the SEPCQ was completed by a total of 749 medical students from two medical schools and 101 physicians from two university hospitals. The SEPCQ was evaluated in several stages involving several of the commonly recommended analytical approaches [56], and the results indicated sound psychometric properties.

### Factor structure

Exploring the possible underlying factor structure, we identified three preliminary factors consisting of a total of 27 items: 1) Exploring the patient perspective, 2) Sharing information and power, and 3) Dealing with communicative challenges. From both a statistical and a content-based perspective, the three factors appeared to be valid subscales covering core aspects of patient-centeredness self-efficacy. The initially defined domain of “viewing the patient’s problem from a bio-psycho-social context” was the only domain, which was only sparsely covered (1 item in Factor 2). Using data from an independent sample, the identified 3-factor model was supported by the confirmatory factor analysis (CFA) showing good fit for the included commonly recommended fit indices. Furthermore, the internal consistencies were generally high across different samples of students and physicians. The main adjustment made to the final SEPCQ-27 was changing the original 7-point Likert scale format with the mid-point of “neither/nor”, which seemed to result in low item difficulties and somewhat positively skewed distributions of Factor 1 and 2, to a 5-point Likert scale. This adjustment appeared to solve this issue and resulted in normally distributed factor scores in subsequent samples.

#### Measurement and response bias

Differential item function (DIF) [47] for an item could indicate measurement bias, i.e., that individuals from different groups with the same underlying ability (latent trait) differ in their likelihood to respond in a certain way to the item. Studies suggest that female physicians engage in more patient-centered communication than male physicians [57], and as it would be important to know whether such differences stem from measurement bias or true gender differences in patient-centeredness self-efficacy, we therefore chose to evaluate possible gender-related measurement bias. Using two different approaches, there seemed to be no signs of DIF for items in Factor 1 and 2. Only two items from Factor 3 showed some indications of DIF with item 25 favoring men (“*I am confident that I am able to stay focused on what is best for the patient if there is a professional disagreement about the diagnosis and treatment”*) and item 26 favoring women (“*I am confident that I am able to avoid letting myself be influenced by preconceptions about the patien*t”). However, as the content of both items appeared to be of importance to patient-centeredness self-efficacy and the values only just exceeded the criteria for DIF, we retained the two items in Factor 3. Response bias is the tendency to respond in an anticipated (socially desirable) manner to certain items [58]. Although our results suggested some degree of social desirability for Factor 1 and 3, but not for Factor 2, it is not clear to which degree this reflects the susceptibility of items to self-deception or impression management among the respondents [59]. The risk of response bias applies to many types of questionnaires, and that some of the attitudes and beliefs concerning patient-centeredness may be somewhat susceptible to social desirability, does not necessarily challenge the validity of the instrument.

#### Construct validity

To provide preliminary evidence for the validity of the SEPCQ, we had, prior to the data collection, stated several hypotheses concerning associations between SEPCQ scores and a number of background variables and additional measures.

Our results generally supported the hypothesized positive associations between SEPCQ scores and age and experience. Older students and physicians had higher scores and years of experience were, independently of age, associated with higher patient-centeredness self-efficacy. The association with age and experience was weakest for Factor 1 (Exploring the patient perspective) and strongest for Factor 3 (Dealing with communicative challenges), suggesting that insight and confidence in dealing with the emotional challenges and conflicts (e.g., item 23 and 24) and disagreement with colleagues (item 25) requires more experience than does confidence in the ability to establish a constructive relationship with the patient (e.g., item 2).

Female medical students have been found to value patient-orientation and communication more than male students [60] and, based on previous studies of physicians [57], we therefore expected that women would score higher than men. Our data, however, did not support any robust association with gender. The lack of an association may reflect a true finding, rather than lack of gender sensitivity of the instrument, as supported by a more recent meta-analysis [61] failing to show clear gender differences in communicative style.

As stress and burnout in residents has been associated with reduced empathy and lower reported patient-centeredness [25], we expected lower levels of medical student well-being, i.e., symptoms of distress and burnout, to be associated with lower SEPCQ scores. This was confirmed by the inverse correlations found between medical student well-being and SEPCQ scores. Well-being scores did, however, not predict pre-post course changes in SEPCQ scores, and the associations between well-being and patient-centeredness are likely to be complex and bi-directional [26].

Self-regulated learning theory predicts that motivational factors such as intrinsic motivation and self-efficacy for learning are important predictors of learning outcomes [62]. Based on previous findings [63–65], we therefore expected that the students’ motivation to learn (CRMTL) and their pre-course self-efficacy (CRSE) with respect to their ability to learn the skills taught in a course in peer-supervision and communication would predict the learning outcome, i.e. changes in their scores on the SEPCQ. In accordance with our hypotheses, level of motivation to learn the skills taught in the course was a significant, independent predictor of SEPCQ change-scores after the course. This suggests the importance of intrinsic motivation for learning patient-centeredness-related skills and, indirectly, provides further support for the construct validity of the SEPCQ.

Finally, the responsiveness of the SEPCQ was supported by increased scores from before to after completing the course in peer supervision and communication. As the effects of the course were not evaluated in a controlled trial, we cannot be certain to which degree the changes are directly related to participating in the course. However, this interpretation finds support in the results showing that physicians who previously had participated in a standard course in communication had higher SEPCQ scores than physicians who had not.

## Conclusions

Taken together, our results indicate that the SEPCQ-27 is a reliable and valid instrument for assessing patient-centeredness self-efficacy in both medical students and physicians. The SEPCQ-27 could be a useful instrument for evaluating patient-centeredness self-efficacy in various contexts, e.g., when evaluating outcomes of communication training courses for medical students and physicians and when studying associations with physician well-being and job-satisfaction. The predictive validity of the SEPCQ remains to be evaluated in future research, e.g., in prospective studies of whether patient-centeredness self-efficacy can predict actual medical student or physician communicative behaviors and patient outcomes such as satisfaction and mental and physical health outcomes.

## Abbreviations

CDR: Combined Decision Rule
CFA: Confirmatory Factor Analysis
CFI: Comparative Fit Index
CRMTL: Course-Related Motivation to Learn Questionnaire
CRSE: Course-Related Self-Efficacy Questionnaire
DIF: Differential Item Function
DMA: Danish Medical Association
ETS: Educational Testing Service
IRT: Item-Response Theory
KR-20: Kuder-Richardson Formula 20
MCSDS: Marlowe Crowne Social Desirability Scale
MH: Mantel-Haenszel
MOT: Medical Outcomes Trust
MSLQ: Motivated Strategies for Learning Questionnaire
MSWBI: Medical Student Well-Being Index
NNFI: Non-Normed Fit Index
SEPCQ: The Patient-Centeredness Self-Efficacy Questionnaire
QAS: Question Appraisal System
RMSEA: Root Mean Square Error of Approximation
S-B-Chi Square: Satorra-Bentler Scaled Chi-Square
SRMR: Standardized Root Mean Square Residual
