# Erratum to: The self-efficacy in patient-centeredness questionnaire – a new measure of medical student and physician confidence in exhibiting patient-centered behaviors

**DOI:** 10.1186/s12909-015-0454-7

**Published:** 2015-10-08

**Authors:** Robert Zachariae, Maja O’Connor, Berit Lassesen, Martin Olesen, Louise Binow Kjær, Marianne Thygesen, Anne Mette Mørcke

**Affiliations:** 1 Unit for Psychooncology and Health Psychology, Department of Oncology, Aarhus University Hospital and Department of Psychology, Aarhus University, Bartholins Allé 9, Aarhus, DK8000 Denmark; 2 Center for Teaching and Learning, School of Business and Social Science, Aarhus University, Fuglsangs Alle 4, Aarhus, 8210 Denmark; 3 Centre for Health Sciences Education, Aarhus University, INCUBA, Palle Juul-Jensens Boulevard 82, bld. B, Aarhus, 8200 Denmark; 4 Faculty of Health, University of Southern Denmark, J.B. Winsløws Vej 19, 3, Odense, 5000 Denmark

## Erratum

Unfortunately the original version of this article [1] contained a mistake. During peer-review, the abbreviation of the questionnaire was changed from ‘PCSEQ’ to ‘SEPCQ’ throughout the article. However, this update was not implemented in Fig. 1, which displays the incorrect abbreviation and is inconsistent with the rest of the article. The figure caption was not affected by this mistake.

Figure 1 should have appeared as follows:

**Fig. 1:**
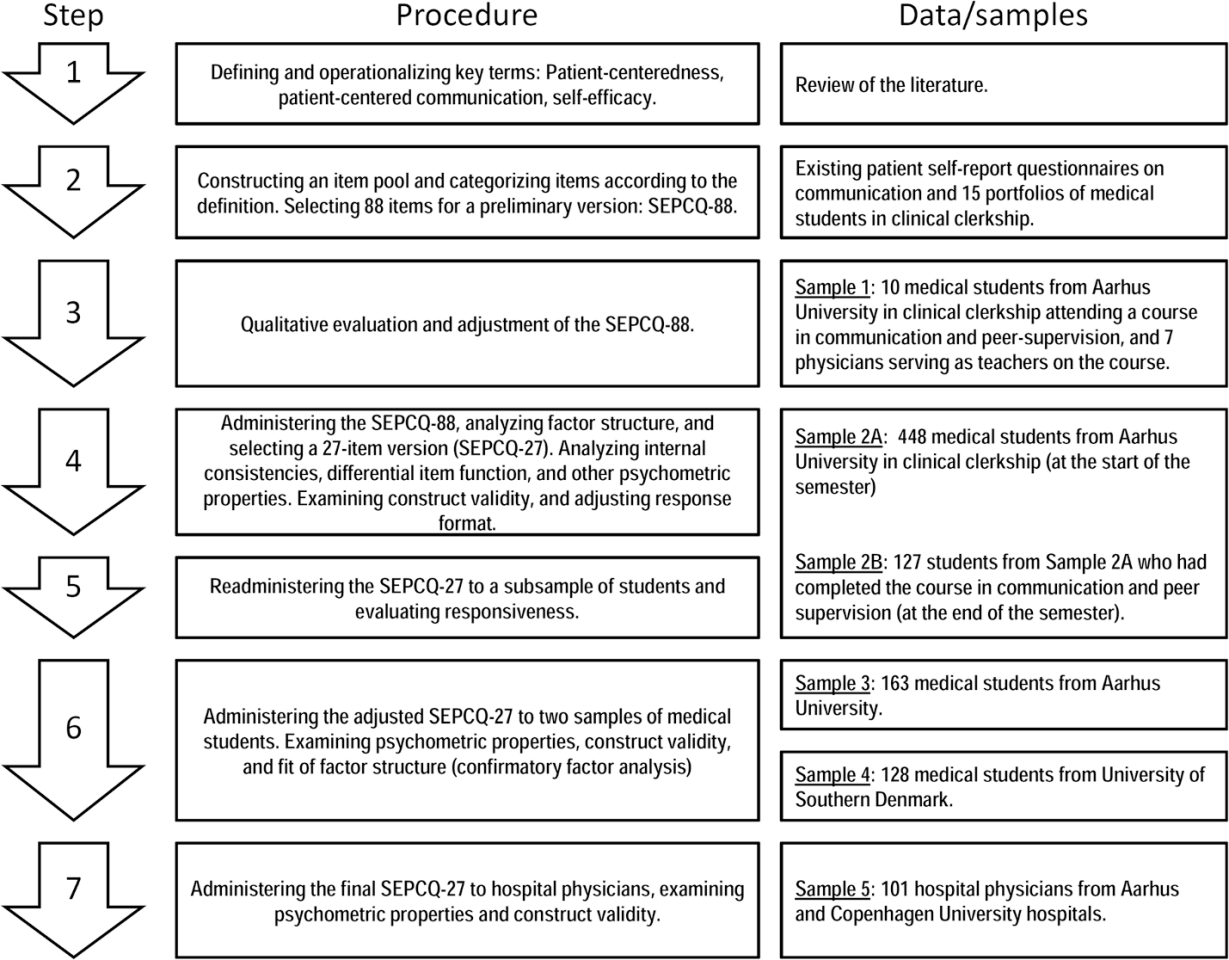
Overview of the development and validation procedure for the Patient-Centeredness Self-Efficacy Questionnaire (SEPCQ)
